# Health-related fitness in medical students: a curricular intervention in Bogota, Colombia

**DOI:** 10.1186/s12889-024-17748-y

**Published:** 2024-01-29

**Authors:** Diana C. Paez, Sebastián Cortés-Corrales, Mario A. Jimenez-Mora, Agustín Gutiérrez, Carlos M. Arango-Paternina, John Duperly

**Affiliations:** 1 School of Medicine, Universidad de los Andes, Bogotá, Colombia; 2 Department of Economics, University of Birmingham, Birmingham, UK; 3 Department of Social Medicine, Universidad de los Andes, Bogotá, Colombia; 4 Instituto Universitario de Educación Física y Deportes, Universidad de Antioquia, Medellín, Colombia; 5 Institute of Exercise Medicine and Rehabilitation, Fundación Santa Fe de Bogotá, Bogotá, Colombia

**Keywords:** Curriculum, Intervention, Lifestyles, Health-related fitness, Physical activity

## Abstract

**Objective:**

To evaluate the impact of a curricular intervention to promote health-related fitness (HRF) among medical students in Bogota, Colombia.

**Method:**

The study was conducted between May 2014 and December 2015 as part of the medical physiology course, in which 208 medical students were enrolled.The curricular intervention included two lectures on physical activity (PA) and student-led group presentations on the physiological effects of exercise on human physiology. An academic incentive (10% of final grade) was given to students who reported and documented regular PA practice during the semester. This study assessed students’ HRF variables, perceptions of the curriculum intervention, and PA practices using quantitative and qualitative approaches.

**Results:**

55% of the students were female, with a mean age of 19.5 years. Body fat, estimated maximum oxygen consumption (VO2max), handgrip, and sit-up strength showed statistically significant improvements at the end of the intervention. Students reported that PA practice was positively influenced by the curriculum intervention, particularly the academic incentive and the HRF tests. Students reported a wide variety of PA practices, which were mainly done with friends, classmates, or family members. Lack of time was the main reported barrier to PA practice.

**Conclusion:**

The curricular intervention was effective in improving HRF and promoting PA. It remains to be investigated whether these gains are sustained over time.

## Background

The prevalence of chronic non-communicable diseases (NCDs) is on the rise globally, with a pronounced impact in developing nations like Colombia [1]. In Colombia, these persistent NCDs stand as the primary contributors to both illness and death recognized [2]. Global surveillance data show that the NCD pandemic could be prevented by reducing common risk factors, mainly tobacco use, physical inactivity, unhealthy diets, and excessive alcohol consumption [3–5]. Accordingly, international health organisations advocate the control of these key behavioural risk factors through the promotion of healthy lifestyles, including physical activity (PA) [6].

The medical community has a key role to play in promoting healthy lifestyles for the prevention and management of diseases [7]. However, few healthcare providers, such us physicians, nurses, medical assistants, psychologists, and physical therapists, have the appropriate environment, knowledge, self-efficacy and lifestyle medicine skills to counsel patients on prevention [8, 9]. Integration of skills into routine clinical practice remains a challenge [10–12]; partly due to infrequent and inadequate training during medical education. Medical schools reported including PA [13, 14] or exercise prescription [15] as part of the curriculum. However, the medical curriculum is insufficiently focused on prevention, is rarely evaluated, and is occasionally adopted [16].

Strategies to integrate PA into lifestyle medicine curricula are needed for current and future physicians to effectively promote behaviour change in patients [17, 18]. Furthermore, not many medical education models promote the adoption of active lifestyles among students. This is important because studies have shown that, in addition to knowledge, personal PA behaviours and physical fitness levels positively influence physicians’ PA counselling practices [19–24]. Knowledge of exercise and fitness provides medical students with a solid foundation in exercise physiology, allowing them to better understand the physiological responses in terms of benefits and adaptations to exercise [25]. This knowledge would enhance physicians ability to provide PA recommendations, and write PA or exercise prescriptions to patients.

There is some encouraging evidence that PA curricular interventions in medical schools have been successful in increasing students’ physical fitness [26], personal PA habits, knowledge, skills, and self-efficacy [27]. However, efforts are needed to adapt and evaluate feasible interventions that can be easily integrated in LMICs. Therefore, the aim of this study is to evaluate the impact of a curricular intervention focused on improving health-related fitness (HRF) and PA knowledge, attitudes, and behaviours among second-year medical students at the Universidad de los Andes in Bogota, Colombia.

## Methods

### Population, setting and study design

A mixed methods intervention design was conducted between May 2014 and December 2015 to assess the impact of an intervention to improve HRF in second year medical students. This design is useful for exploring in more detail the impact of an intervention and participants’ perceptions of [28]. In this case, the integration of the quantitative and qualitative research components occurred after the intervention. The intervention was embedded in the compulsory medical physiology course across three cohorts of students. Data were available from 208 enrolled medical students (68 in the second semester of 2014, 88 in the first semester of 2015 and 52 in the second semester of 2015) at the Universidad de los Andes in Bogota, Colombia.

The outcomes assessed were students’ HRF variables, their perceptions of the curriculum intervention and PA practices.

*Medical Physiology course description*: The course provided second year (i.e. fourth academic semester) medical students with a basic knowledge of how the human body functions to further understand disease processes and therapeutic approaches. The course covered molecular and cellular physiology and major physiological topics such as cardiopulmonary, neuromuscular, endocrine, gastrointestinal and renal systems. The course consisted of 60 h during the 20-week academic semester. A numerical scale from 0 to 5 was used for grading. Course assessment was based on the following: three exams (70% of total grade), quizzes (10% of total grade) and group presentations (10% of total grade). In addition, 10% of the total course mark was allocated to students’ self-assessed personal commitment to PA. A total of 10% of the questions in the exams and quizzes were dedicated to exercise physiology.

### Quantitative component

A quasi-experimental study design was conducted. In total, 12 of the 60 h (20%) of the medical physiology course were spent on the curriculum intervention. The curriculum intervention consisted of lectures on muscle contraction and exercise physiology for a total of 6 h during the academic semester. In addition, 3 h were allocated to student-led group presentations on the effects of healthy habits (i.e. exercise, diet, tobacco cessation) on specific pathophysiological processes. Basic HRF assessments were administered at the beginning (week 1) and at the end of the course (week 20), and took a total of 3 h to complete.

#### Group presentations

Students were asked to form groups of 8. Each group was randomly assigned an academic topic on the prevention and/or treatment of disease through the promotion of PA, to be researched through a literature review. At the end of the course, students were asked to submit an abstract (500 words) and give a 10-minute academic presentation to classmates, the professor and experts in the field. The presentations were evaluated by the members of the evaluation panel according to a pre-established rubric. The pre-established rubric comprises five key criteria (1. Quality of research and content, 2. Structure and organization of the presentation, 3. Quality of the presentation, 4. Ability to comprehend the topic and respond to questions from the audience and the evaluation panel, and 5. Group collaboration and contribution).

#### Academic incentive for PA practice

An important component of the curriculum intervention was an academic incentive to encourage students to practise PA during the semester. Students received support from teaching assistants and resources available at the university. For example, students were encouraged to attend the sports centre free of charge (i.e. the sports centre serves as a free resource available to the student community) and those who attended were trained by sports professionals. The 10% of the final grade, as described above, was agreed between the professor and the student independently of the HRF test results. No student received a grade lower than 3 out of 5. Students were also told that their answers would not affect their final grade.

#### Outcome variables

HRF variables were assessed before and after the curricular intervention. All measurements were carried out at the Universidad de los Andes Sports Centre according to a standardised protocol. A detailed manual of procedures was developed by the research group and was reviewed by the staff prior to the measurements. The staff consisted of research assistants and trained medical students. After basic screening for PA readiness (PAR-Q and YOU) [29], students received instructions (e.g., use of sports clothing, hydration) and recommendations for possible contraindications (i.e., acute illness, fever, chest pain). None of the students had any contraindications.

Weight and height were measured in lightly dressed students with shoes removed. Body weight was measured using calibrated electronic scales (Beurer Bg 21 Tanita Omron) and height using an acrylic stadiometer. Body mass index (BMI) was calculated from weight and height (kg/m2) and categories (i.e. underweight, normal weight, overweight and obesity) were calculated according to the World Health Organization standards (WHO, 2000) [30]. A handheld device was used to measure body fat percentage using a bioelectrical impedance device (Omron HBF-306 C).

Cardiorespiratory fitness was assessed using the 20-metre multistage shuttle run test (20-MST), previously validated for the study population [31]. Students were required to walk or run between two lines 20 m apart at a pace announced by recorded tones at standard intervals. For any given participant, the test was terminated if the participant failed to reach the line in time for the beep on two consecutive occasions. Heart rates were recorded continuously throughout the test (Polar heart rate monitor, model FT7). The last stage completed was recorded and then used to estimate maximum oxygen consumption (VO2max) according to the equation of Leger et al. [32].

Grip strength was assessed using the JAMAR® hand dynamometer (JAMAR, 5030J1), which was calibrated prior to the measurements. Students were asked to assume a seated position and grasp the dynamometer with the dominant hand, with the shoulder adducted and neutrally rotated, the elbow flexed at 90º, the forearm in neutral position, and the wrist between 0º and 30º dorsiflexion and between 0º and 15º ulnar deviation. Students positioned the instrument so that it fit comfortably in their hands. Once the student was in the correct position, the staff gave a verbal instruction to ‘squeeze as hard as possible’ and apply maximum force. The test was repeated three times. Maximum grip force was recorded in kilograms [33].

Abdominal muscle endurance was measured as the maximal number of sit-ups in 30 seconds. Students were asked to lie on a mat with their knees bent at right angles, their feet flat on the floor and held down by a partner. Their fingers had to be interlocked behind their heads. On the command ‘go’ the participants had to touch their knees with their elbows and then return to the floor. The maximum number of repetitions in 30 s was recorded.

#### Statistical analysis

Means and standard deviations were calculated for demographic, body composition and fitness variables for all participants, stratified by sex. T-tests assuming equal and unequal variance (according to F-test results) were performed to analyse baseline differences by sex. Two-tailed paired Student’s t-tests were used to compare body composition and fitness variables before and after the intervention in men and women separately. The effect of the intervention was calculated using a potential outcome framework using the mean difference between pre and post scores [34]. This methodology considers the pre-test measure as the contra-factual (i.e., control) for each student. Five multilevel mixed-effects generalized linear regression models were run to estimate the effect of the intervention on body composition, cardiorespiratory fitness and muscular strength and endurance. An individual mixed effect was used in the regression analyses to control for the individual inherent time-invariant characteristics (i.e., confounds) that may affect the students’ progress during the semester. Specifically, we use a series of linear regression models to estimate the relationship between our dependent variables and various factors, taking into account random effects at both the cohort and student levels. In this way, we control for all time invariant-confounds that may arise by the cohort and students’ idiosyncratic characteristics that might affect our variables of interest. For each of our dependent variables, including BMI, body fat percentage, VO2max, handgrip strength and sit-up strength, we adopt the following specification.

$$ \begin{gathered}{y_{ict}} = {\beta _0} + {\beta _1}Interventio{n_t} + {\beta _2}Gende{r_i} \hfill \\\,\,\,\,\,\,\,\,\,\,\,\,\,\, + {\beta _3}Interventio{n_t}*Gende{r_i} + {\gamma _i} + {\delta _c} + { \in _{ict}} \hfill \\ \end{gathered}$$

where $$ i$$ refers to each student, while $$ t$$ denotes the period, i.e. before and after the intervention. The multivariate regression model includes the following regressors $$ Interventio{n}_{t}$$ a dichotomous variable that takes the value 0 before the intervention and 1 after the intervention, and $$ Gende{r}_{i}$$ another dichotomous variable based on the gender of each student. Here, female students are coded as 1, while male students are coded as 0. In addition, we account for the random effects of each student and cohort using the normal standard random variables $$ {\gamma }_{i}$$ and $$ {\delta }_{c}$$, respectively. For the regression with the dependent variable VO2max, we included peak heart rate as a control as it is a potential confounder. Statistical analyses were performed using Stata 12 and a 95% confidence interval for hypothesis testing (StataCorp LP, College Station, TX, USA).

### Qualitative component

An interpretive descriptive qualitative design [35] was used to assess students’ PA practices and their perceptions of the curriculum intervention. Data were collected through self-report assessment and semi-structured interviews over two academic semesters (II-2014 and I-2015).

At the end of the course, students were asked to submit self-reports detailing their level of PA adherence, perceptions of sport skills, and personal experiences of engaging in PA. These self-reports were submitted in written form. In addition, students were encouraged to provide supporting documentation to supplement their reports, including photographs and sports centre attendance records.

The interview process was conducted by a diverse team of four trained researchers specialising in qualitative data collection methods (i.e. an anthropologist, a psychologist, an economist and a physiotherapist). Several themes were explored during the interviews, including the students’ motivations and their support network (i.e. friends and family members). Students were also asked to provide insights into the benefits and barriers they encountered when engaging in PA, their preferred types of PA, the locations where they preferred to engage in PA, and the people with whom they usually engaged in PA. In addition, the interviews proved valuable in understanding students’ perceptions of the curriculum intervention and in identifying the factors influencing behaviour change over the semester.

*Participants*: Purposive sampling was used to select participants. The selection criteria were medical students enrolled in the medical physiology course who participated in the physical fitness tests. During II-2014, interviews were conducted with students (*n* = 6) as a pilot interview guide for clarity and applicability of questions. During the academic term I-2015, 36 students participated in the semi-structured interviews: 22 students were included in the pre-intervention and 14 students in the post-intervention.

#### Data analysis

The interviews were audio-recorded, transcribed verbatim by the interviewers and analysed using ATLAS.ti 7. The data collected were coded into categories and then analysed according to suggested procedures [36]. Theoretical saturation was achieved at all interview stages [37]. Notes were translated into English by the bilingual research staff of the Universidad de los Andes.

### Ethical aspects

This study was approved by the Ethical Review Committee of the University of Los Andes (Act No. 538/2015). Students’ participation in the physical fitness tests, self-reports, and interviews was voluntary. Those who agreed to participate in the study signed an informed consent form.

## Results

A total of 208 students were enrolled in the physiology course, 68 in II-2014, 88 in I-2015 and 52 in II-2015. One hundred and ninety-one students participated in the pre-intervention physical fitness tests and 172 students participated in the post-intervention physical fitness tests. One hundred and sixty-six students completed information on all dependent variables (i.e. BMI, body fat, estimated VO2max, handgrip strength and sit-up test) in both the pre- and post-tests. Of the participating students, 58% were female with a mean age of 19.5 years. As expected, significant differences in body composition variables and fitness test performance were observed between males and females at baseline (Table 1).

**Table 1:** Demographic characteristics, body composition, and baseline fitness tests in second year medical students (*n* = 166)

Variable	Male (*n* = 70)	Female (*n* = 96)	*p*-value (Paired t test)
	Mean (SD)	Mean (SD)	
Age (years)	19.7 (1.8)	19.4 (1.3)	0.337
Weight (kg)	68.4 (10.5)	58.6 (9.9)	**< 0.001**
Height (cm)	174.9 (6.0)	162.3 (6.3)	**0.003**
Body mass index (kg·m^-2^)	22.3 (2.7)	22.0 (3.8)	0.558
Body fat (%)	16.0 (6.4)	25.0 (5.7)	**< 0.001**
Estimated VO2max (ml·kg^-1^·min^-1^)	40.5 (5.1)	31.1 (3.7)	**< 0.001**
Peak heart rate (bpm)	192.2 (11.4)	179.1 (19.6)	**< 0.001**
Handgrip strength (kg)	43.2 (8.3)	26.7 (4.3)	**< 0.001**
Sit up strength (# sit ups in 30 s)	26.1 (5.3)	20.8 (3.9)	**< 0.001**

The sample was homogeneous within the three academic semesters, there were no significant differences for age, BMI, estimated VO2max or sit-up test. However, for the sit-up test, students from different semesters showed slight differences with a variation of 2 sit-ups.

Table 2 shows the means and standard deviations for the body composition and fitness variables for male and female participants. There were no statistically significant changes in BMI or peak heart rate on the Leger test between the two assessments. However, statistically significant differences were observed in body fat percentage, estimated VO2max, handgrip strength and sit-up strength over time.

**Table 2:** Pre and post levels of the body composition and fitness tests in second year medical students (*n* = 166)

Variable	Male (*n* = 70)			Female (*n* = 96)		
	Pre Mean (SD)	Post Mean (SD)	*p*-value (Paired t test)	Pre Mean (SD)	Post Mean (SD)	*p*-value (Paired t test)
Body mass index (kg·m-2)	22.3 (2.7)	22.5 (2.5)	0.131	22.0 (3.8)	22.0 (2.9)	0.943
Body fat (%)	16.3 (6.3)	14.9 (5.6)	**< 0.001**	25.0 (5.7)	24.2 (5.2)	**0.035**
Estimated VO2max (ml·kg-1·min-1)	40.7 (5.1)	43.8 (5.3)	**< 0.001**	31.1 (3.7)	34.6 (3.8)	**< 0.001**
Peak heart rate (bpm)	192.0 (11.3)	193.8 (10.4)	0.197	178.7 (20.2)	181.9 (16.6)	0.284
Handgrip strength (kg)	43.0 (8.1)	45.7 (9.8)	**0.001**	26.7 (4.3)	28.5 (5.3)	**< 0.001**
Sit up strength (# sit ups in 30 s)	26.1 (5.3)	28.7 (4.4)	**< 0.001**	20.8 (3.9)	24.2 (3.6)	**< 0.001**

In the regression adjusted models, women had higher body fat and lower estimated VO2max, handgrip strength and sit-up strength at baseline (Table 3). After the intervention, body fat decreased by 1.54% [CI: -2.42 to -0.67; *p*-value < 0.01], estimated VO2max improved by 3.22 ml·kg-1·min-1 [CI: 2.43 to 4.01; *p*-value < 0. 01], handgrip strength increased by 2.48 kg [CI: 1.03 to 3.93; *p*-value < 0.01], and the 30-second sit-up test increased by 2.84 sit-ups [CI: 2.00 to 3.68; *p*-value < 0.01]. Conversely, no differences in BMI were observed after the intervention. The effect of the intervention on all dependent variables did not differ by sex.

**Table 3:** Impact of curricular intervention on measures and physical fitness tests in medical students (*n* = 166)

	Body mass index (kg·m-2)	Body fat (%)	Estimated VO2max (ml·kg-1·min-1)	Handgrip strength (kg)	Sit-up strength (#sit-up in 30 s)
Intervention’s impact	0.16	-1.54***	3.22***	2.48***	2.84***
	[-0.33, 0.64]	[-2.42, -0.67]	[2.43, 4.01]	[1.03, 3.93]	[2.00, 3.68]
Sex (reference Male)	-0.39	8.77***	-6.31***	-16.11***	-5.30***
	[-1.24, 0.46]	[7.19, 10.34]	[-9.75, -2.88]	[-18.08, -14.14]	[-6.53, -4.07]
Sex*intervention	-0.11	0.54	0.38	-0.65	0.65
	[-0.76, 0.54]	[-0.63, 1.70]	[-0.67, 1.42]	[-2.57, 1.27]	[-0.47, 1.77]
Peak heart rate			-0.00		
			(-0.03, 0.02)		
Constant	22.44***	16.21***	39.96***	43.07***	25.96***
	[21.79, 23.08]	[15.02, 17.40]	[34.22, -45.71]	[41.44, 44.69]	[24.66, 27.26]

Confidence interval in square parentheses

*** *p* < 0.01, ** *p* < 0.05, * *p* < 0.1

### Curriculum intervention

Fifty-seven per cent of the students surveyed (8 out of 14) said that the curriculum - lectures, case observation and discussions - had a positive impact on their PA practice.

*“I think it is important for us to be motivated to be physically active (…). As the problems associated with a sedentary lifestyle have become more apparent during the lectures, we are more aware of the need to improve our fitness” (Male student, 20 years)*.

The academic incentive was a motivational strategy for most students to practice PA, start a routine or exercise during the semester, and maintain active behaviours.

*“For a lot of us, the academic incentive is very important, the university looks at you for your grades, so it’s important to try to get the highest grade possible. Anything that improves the average grade is really motivating for me (…), I would say it contributed to 50% of my decision to be more active” (Male student, 21 years old)*.

Similarly, students reported that the physical fitness tests encouraged them to set goals and adopt healthy behaviours during the semester.

*“The fitness test is used to evaluate yourself; you set goals at the beginning of the semester and are encouraged to achieve them. (Male student, 19 years old)*

### PA practice

Only 8 out of 22 students (36.4%) interviewed before the intervention reported practising PA (150 min of moderate intensity PA or 75 min of vigorous intensity PA per week), compared to 12 out of 14 students (85.7%) interviewed after the intervention.

Most students reported practising PA three times a week, with the duration of each session ranging from 45 min to 2 h. Selected types of activities were sports (volleyball, football, tennis, ultimate taekwondo and kickboxing), workouts (TRX, CrossFit, Zumba, Pilates and gym strengthening routines) and aerobic activities such as running and swimming. Walking and cycling were the modes of transport used by students to and within the campus.

Most students used university infrastructure facilities (e.g., sports centre, healthy circuits) for physical activity and participated in PA courses (1-credit electives) offered as part of the degree credits for all academic programmes at the university.

### Student’s external motivations and barriers for PA practice

Motivators such as sharing healthy behaviours with friends and family were important motivators for students to adopt and maintain the behaviour change.

Among students who reported that family members and friends were physically active, 85.7% (12 out of 14) and 35.7% (5 out of 14), respectively, reported engaging in PA after the intervention.

*“During the semester, I started to encourage my family to go outside and do some physical activity. We now play basketball on Sundays” (Female student, 21 years old)*.

Family members and friends played an important role in the adoption of students’ PA habits.

Two elements were identified:

1. previous PA habits of family members,

*“I consider myself lucky to have my father as a role model, he is an excellent professional and an admirable athlete. (…) I grew up with a lifestyle where outdoor activities were plentiful and always involved some kind of physical effort. This made me love sport” (Female student, 21 years)*.

(2) feedback students received from family members after adopting healthy habits

*“I was able to influence some members of my family to exercise, first my mother started walking with me around the neighbourhood, then she was no longer able to walk, but my brother joined me” (Female student, 19 years)*.

Group activities were reported as motivational and social support elements to start and continue healthy habits.

Academic workload and lack of time were the main barriers to PA reported by students, consistent with the decrease in PA practices during the semester (i.e., final exams in November 2014 and May 2015).

*“I don’t exercise, I tried to exercise a month ago but I couldn’t keep up the habit (…) Being at university is hard, I don’t have time and also I’ve never had a routine” (Female student, 19 years old, 2015)*.

### Healthy benefits

Improvements in fitness, appearance, stress, anxiety, sleep, smoking cessation, diet and weight control were the most commonly perceived benefits of PA by students.

*“I like to exercise because it gives me energy and I do it to improve my fitness, (…) I think it is important to maintain healthy habits” (Female student, 18 years)*.

Students also reported that the health benefits of PA were associated with improved attitudes to preventive care for future medical practice.

*“As a future doctor, I feel morally obliged to recommend physical activity because I now know its physical and mental health benefits (…) I want my patients to adopt and maintain a healthy lifestyle” (Male student, 19 years)*.

## Discussion

We found that a one-semester curricular intervention was effective in improving the HRF variables studied in medical students. Cardiorespiratory fitness, body fat and muscular fitness improved significantly from baseline. These results are consistent with findings from intervention studies of similar duration. For example, Project Active, a randomised trial (*n* = 235) comparing two interventions in a sedentary middle-aged adult population, showed an increase in estimated VO2max of 1.58 ml·kg-1·min-1 (95% CI: 0.93 to 2.22) for the lifestyle intervention group and 3.64 ml·kg-1·min-1 (95% CI: 2.98 to 4.29) for the structured exercise programme group after 24 weeks of intervention [38]. Compared to our study, we found a similar increase in estimated VO2max (3.11 ml·kg-1·min-1) after the 20th week of intervention. In addition, a systematic review [39] of health behaviour change programmes found a median net increase in estimated VO2max of 6.3% (interquartile range, 5.1–9.8%) in adult participants. The present study calculated a net increase in estimated VO2max of 8.9%, which is consistent with the data from the available studies.

Regarding changes in body composition, the Project Active study found a -1.4% (95% CI: -1.8 to -0.9) decrease in body fat percentage for the lifestyle intervention group and a -1.7% (95% CI: -2.1 to -1.2) decrease for the structured exercise programme group [40], similar to the decreases found in this study. Contrastingly, we did not find significant changes in BMI after the intervention. The unchanged BMI levels highlights the fact that the intervention did not lead to changes in body weight. However, this finding does not rule out beneficial changes in body composition, which are not considered by the BMI formula. The significant decrease in body fat percentage that we observed, along with the increase in muscle fitness and the stable BMI, indicate an increase in fat-free mass. Several studies have reported the same favorable changes in body composition (decreased fat mass and increased fat-free mass) with minimal or no change in BMI after controlled aerobic and resistance training programmes [41] usually lasting 15 to 20 weeks; these changes are usually small in magnitude [42]. Furthermore, weight loss was not expected in this population with healthy baseline BMI. Evidence suggests that high amounts of PA (≥ 250 min·week-1) plus dietary changes involving energy restriction are required to achieve weight loss [43]. According to the apparent dose-response relationship between PA and weight loss, PA levels between 150 and 250 min·week-1 are associated with weight maintenance [43], which is consistent with our results.

In terms of muscular fitness, handgrip strength and abdominal muscle endurance increased significantly from baseline. On the one hand, resistance training programmes alone or in combination with aerobic exercise have been reported to increase handgrip strength in healthy medical students [44]. On the other hand, several studies lasting 7 to 24 weeks have reported improved sit-up performance after traditional sit-up/curl up programmes [45, 46], core stabilisation exercise programmes [46], integrated resistance training programmes with functional movement patterns [47], upper/total body resistance training programmes [48] and unstable resistance training programmes [49], but not after aerobic training alone [48]. Therefore, the students’ reports of participation in both resistance and aerobic PA training are consistent with the results of the physical tests.

There is a paucity of studies on PA promotion among medical students. A systematic review [27] included 11 studies that evaluated the effectiveness of 10 PA counselling programmes for undergraduate medical students. There are several similarities between these programmes and our study. First, all studies in this area were quasi-experimental, reflecting trends and limitations in medical education research. Second, most interventions were incorporated into already established courses; as in this curricular intervention, the PA promotion strategy was incorporated into the medical physiology course. Thirdly, all studies included experiences or didactics of PA as a health behaviour. Fourthly, half of the studies asked students to report on their personal experience of PA. Three of the included studies asked students to change their own healthy habits, and only Frank et al. [50] required this habit to be PA. In addition, only the latter included pre- and post-intervention assessments.

Behavioural change theories and socio-ecological models are essential for effectively understanding and planning interventions to promote healthy lifestyles [51]. Bauman et al. propose individual, interpersonal and environmental dimensions as determinants of PA [52]. The qualitative methodology used in this study provided complementary information on the three dimensions described.

Our intervention resulted in the vast majority of students meeting the recommended amounts of PA, much more than the average medical student in Colombia [23] and higher than the PA levels of physicians and medical students in the United States [53–55] and the United Kingdom [56].

We found that students preferred to participate in group sports or activities, and that having an active family member or friend was an important motivator for engaging in PA. These findings add to the existing literature linking social support with increased PA in LMIC [57, 58], and specifically among medical students [15]. Promoting group sports and activities (such as aerobics classes, running clubs or football teams) may be an important element to include in future interventions for medical students.

Previous reviews have shown that lifestyle education for medical students needs to be an important part of the curriculum to have a positive impact on PA counselling [15, 27, 59]. In the present study, we showed that a 20-week intervention can be effective in promoting healthy lifestyles in relation to PA in second-year medical students. In a previous study, a lifestyle course delivered within 24 h was shown to be ineffective in improving health behaviours in medical students [60]. The frequency of PA among students in this study (i.e. an average of 3 days per week) is consistent with recent recommendations for health and fitness benefits. The position statement of the American College of Sports Medicine also states that healthy adults, such as our students, whose goal is to improve physical fitness and health, can also benefit from more intense advanced training techniques [61].

Our study found that the academic incentive was an effective motivator for compliance, similar findings have been documented [62]. However, it remains to be understood whether such an incentive produces lasting changes. In addition to improvements in HRF variables, our intervention provided perceived benefits such as a reduction in stress and an improvement in sleep patterns according to student self-reports. Previous research has shown that medical students have poorer mental health and perceive more stress than the general population [63, 64]. Research among undergraduates has found a negative relationship between PA levels, perceived stress and mental health [65, 66], which is consistent with our current findings. In addition, more active medical students were less likely to report symptoms of depression and anxiety [67]. Thus, these types of physical activity programmes appear to be an effective way to help students cope better with the stressors inherent in medical school, but more research is needed to better assess this claim [68].

Finally, one of the most commonly cited barriers to engagement in PA is lack of time, particularly among medical students [69–71]. Time constraints are particularly prevalent among medical students when considering stressful factors such as academic pressure, social issues and financial difficulties, which have been found to be higher compared to other undergraduate academic programmes and as academic careers progress [72, 73]. Nevertheless, time constraints for PA practice among medical students have been linked to a lack of adequate time management in similar studies [74, 75]. These findings highlight the importance of addressing time constraints by, for example, encouraging medical students to use more active transport, engage in more vigorous activities and make use of existing campus infrastructure.

### Strengths and limitations

Strengths of this study include the use of both quantitative and qualitative methods. Objective data such as body composition and fitness test performance were chosen as outcome variables for the quantitative analyses. Limitations include the lack of a control group for intervention comparison. Students’ PA practices and perceptions of the curriculum were based on self-reported data. Findings on the benefits students perceived from PA (e.g. improved sleep patterns and better eating habits) were self-reported and there were no objective measures to confirm such benefits. Finally, the results may not be generalisable to other situations.

## Conclusion

A curricular intervention was effective in improving second year medical students’ HRF variables and promoting personal PA habits. Students were positively influenced by the curricular intervention to promote PA during the semester, which is expected to be maintained over time and to be reflected in favourable attitudes towards health promotion for clinical practice, prevention programmes and policy initiatives. These findings should encourage medical schools to incorporate curricular strategies to promote PA and related knowledge among medical students. Further evaluation is needed to assess long-term effects on HRF variables and personal PA behaviours, as well as counselling practices among medical students.

## Data Availability

The data used and/or analyzed in the current study are available from the corresponding author on reasonable request.

## Abbreviations

PA: Physical activity
HRF: health-related fitness
NCDs: Non-communicable diseases
LMICs: low and middle-income countries
VO2max: maximum rate of oxygen consumption
BMI: Body Mass Index
20-MST: 20-meter shuttle run test
